# Dual Immune Regulatory Roles of Interleukin-33 in Pathological Conditions

**DOI:** 10.3390/cells11203237

**Published:** 2022-10-14

**Authors:** Han Guo, Elhusseny A. Bossila, Xinran Ma, Chenxu Zhao, Yong Zhao

**Affiliations:** 1 State Key Laboratory of Membrane Biology, Institute of Zoology, Chinese Academy of Sciences, Beijing 100101, China; 2 University of Chinese Academy of Sciences, Beijing 101499, China; 3 Biotechnology Department, Faculty of Agriculture Al-Azhar University, Cairo 11311, Egypt; 4 Beijing Institute for Stem Cell and Regeneration, Beijing 100101, China

**Keywords:** IL-33, ST2, immune system, inflammation, alarmin, disease, organ transplantation

## Abstract

Interleukin-33 (IL-33), a member of the IL-1 cytokine family and a multifunctional cytokine, plays critical roles in maintaining host homeostasis and in pathological conditions, such as allergy, infectious diseases, and cancer, by acting on multiple types of immune cells and promoting type 1 and 2 immune responses. IL-33 is rapidly released by immune and non-immune cells upon stimulation by stress, acting as an “alarmin” by binding to its receptor, suppression of tumorigenicity 2 (ST2), to trigger downstream signaling pathways and activate inflammatory and immune responses. It has been recognized that IL-33 displays dual-functioning immune regulatory effects in many diseases and has both pro- and anti-tumorigenic effects, likely depending on its primary target cells, IL-33/sST2 expression levels, cellular context, and the cytokine microenvironment. Herein, we summarize our current understanding of the biological functions of IL-33 and its roles in the pathogenesis of various conditions, including inflammatory and autoimmune diseases, infections, cancers, and cases of organ transplantation. We emphasize the nature of context-dependent dual immune regulatory functions of IL-33 in many cells and diseases and review systemic studies to understand the distinct roles of IL-33 in different cells, which is essential to the development of more effective diagnoses and therapeutic approaches for IL-33-related diseases.

## 1. Introduction

Interleukin-33 (IL-33), identified about twenty years ago as a new member of the IL-1 family (IL-1F) [1], plays pivotal roles in host innate and adaptive immunity, homeostasis, tissue repair, and responses to environmental stress. The *IL33* gene is located on chromosome 9p24.1 of the human genome and on chromosome 19qC1 of the mouse genome. The human and mouse IL-33 cDNA sequences code 270 and 266 amino acids and produce 30 and 29.9 kDa polypeptides, respectively [1]. Full-length IL-33, also known as IL-1F11, is identical to nuclear factor from high endothelial venules reported in 2003 [2]. The C-terminal domain of IL-33 meets the characteristics of IL-1F, and the N-terminal region contains a non-classical nuclear sequence and a chromosomal binding domain with a homeodomain-like helix–turn–helix motif [3,4]. Human and mouse IL-33 share 52% of an identity at the amino acid level, and recombinant human IL-33 can stimulate mouse lymphocytes as effectively as recombinant mouse IL-33 [5]. Under physiological circumstances, IL-33 is predominately localized in the nucleus, where it binds to chromatin via the tails of histones H2A and H2B and regulates gene expression [4,6]. However, IL-33 is quickly released upon external stimulation and subsequently mediates immune response as an alarmin cytokine, as it is passively released by damaged or necrotic endothelial and epithelial cells [7]. Multiple types of cells, including endothelial cells, epithelial cells, smooth muscle cells, and immune cells, can produce IL-33. Extracellular IL-33 binds to suppression of tumorigenicity 2 (ST2, also called IL-33R or IL-1R4) and its co-receptor IL-1 receptor accessory protein (IL-1RacP, also known as IL-1R3) to mediate biological functions in a variety of physiological and pathological processes [8,9,10]. A number of studies have uncovered the complicated and multiple roles of IL-33/ST2 in physiological and pathological situations, which has attracted wide interest in understanding the significance of IL-33 in diseases, as well as exploring the potential clinical application of approaches that target IL-33/ST2. In the present review, we will summarize the major biological functions of IL-33 and discuss the involvement of IL-33 in various diseases.

## 2. Expression and Release of IL-33

**Figure 1:**
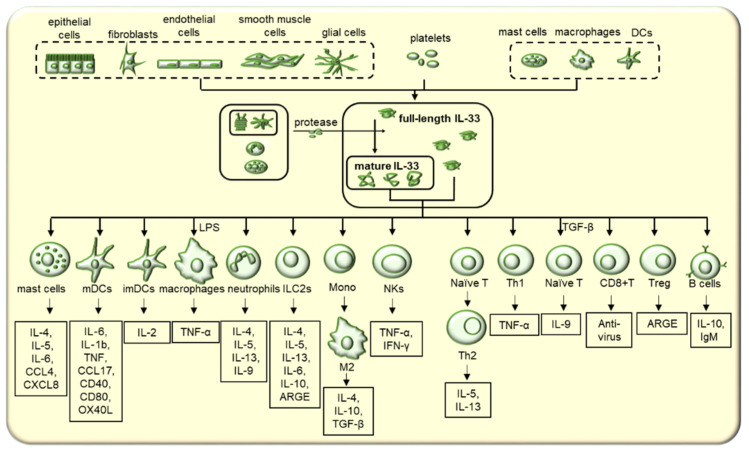
The production and roles of IL-33 in immune responses. Under pathological conditions, full length IL-33 is released by endothelial cells, epithelial cells of barrier tissues such as lung, intestine, skin, and fibroblasts, and also glial cells and astrocytes, smooth muscle cells and platelets and several types of immune cells, including macrophages, dendritic cells (DCs), and mast cells. After cleavage by proteases, highly active IL-33, interacting with ST2, activates different immune cells to generate different cytokines or polarize into the corresponding phenotypes in different pathological conditions.

IL-33 has been mainly detected in endothelial cells, epithelial cells of barrier tissues such as lung, intestines, skin and fibroblasts, and also glial cells and astrocytes in the brain, Müller glial cells in the eyes, and smooth muscle cells. Moreover, it has been reported in platelets and several types of immune cells, including macrophages, dendritic cells (DCs), and mast cells [11,12] (Figure 1), as reviewed by Cayrol et al. [13]. The expression of IL-33 is generally classified into two patterns: constitutive and induced or transient, and the expression pattern determines IL-33 activities. In the steady state, IL-33 is constitutively expressed and located in the nuclei in the form of full length, while cellular damage and necrosis caused by external stimulation enhances IL-33 expression and induces IL-33 maturation [14]. For example, mechanical wounding by scratching or cell necrosis by several cycles of freezing and thawing significantly increased IL-33 expression and release [15]. IL-33 expression is increased in airway epithelial cells of patients with asthma or chronic obstructive pulmonary disease and in mouse type 2 alveolar epithelial cells by pathogens and allergens [16]. In addition, proinflammatory cytokines such as IFN-γ [17], IL-4, and IL-13 [18], and Notch signaling [19] have been reported to increase IL-33 expression.

The expression and regulation of IL-33 partially differ across species. Human endothelial cells, but not mouse, constitutively express IL-33 in the nucleus under baseline conditions [20,21]. IL-33 is constitutively expressed in murine keratinocyte nuclei and is rapidly lost during acute inflammation as an alarmin. In contrast, IL-33 is almost absent from human and porcine keratinocytes in normal skin but can be induced upon acute inflammation, such as by interferon-γ (IFN-γ) [22]. Mouse endothelial cells produce IL-33 only under inflammatory conditions, but mouse epithelial cells constitutively express IL-33, and its expression is rapidly decreased under inflammatory conditions [1,23,24]. Alveolar type II pneumocytes are the major source of IL-33 in murine lungs, whereas airway basal epithelial and endothelial cells are the primary sources of IL-33 in human lungs [25]. Concentrations of IL-33 significantly increased after murine lung epithelial cells were incubated with live or heat-killed *Aspergillus fumigatus* conidia [26], indicating that lung epithelial cells play a vital role in initiating the innate immune response to *Aspergillus fumigatus* infection. In cases of apoptosis and necrosis, IL-33 is released differently. During apoptosis, caspase-3 and caspase-7 hydrolyze and deactivate full-length IL-33 [15,27,28], whereas IL-33 stored in the nucleus is released to act as an alarm element or DAMP during necrosis. In necrosis, full-length IL-33 is directly released into the extracellular space and is cleaved by the proteases to generate mature IL-33 [9].

There are several signaling pathways that participate in regulating IL-33 expression. Apigenin and luteolin suppress IL-33 production in lipopolysaccharide (LPS)-activated microglial cells through the mitogen-activated protein kinase (MAPK), nuclear factor-κB (NF-κB), and signal transducer and activator of transcription 3 (STAT3) signaling pathways [29]. The chromatin remodeling protein BRG1 positively regulates transcription of IL-33 in endothelial cells in a mouse model of ischemia-reperfusion (IR)-induced renal injury [30]. Macrophages and DCs produce IL-33 following toll-like receptor (TLR) stimulation [31]. IL-33 production in mouse macrophages and fibroblasts is regulated by the STAT1 signaling pathway, as well as by the transcription factors interferon regulatory factor 3 and cAMP response element binding protein, after stimulation by TLRs or IFN-γ [32,33]. A recent study showed that c-Jun, a component of activator protein 1 (AP-1), directly regulates IL-33 gene expression by binding to its enhancer depending on focal adhesion kinase-controlled chromatin accessibility [34]. Microparticles induce IL-33 production by macrophages in a Bruton’s tyrosine kinase (BTK)-dependent manner [35], suggesting that both BTK and IL-33 may be promising therapeutic targets in cases of wear debris-induced periprosthetic inflammation. Mast cells produce IL-33 and enhance the local IL-33 concentration of damaged tissues after stimulation by IL-25 and thymic stromal lymphopoietin (TSLP) [36].

The mechanisms for IL-33 release from the cell are currently unclear. As it lacks an endoplasmic reticulum–Golgi secretion signal sequence, it is different from the classical secretory manner. Currently, three pathways for IL-33 release were reported. First, full length IL-33 functioned as an alarmin released in extracellular space after cellular damage or necrosis [16]. Second, DCs of mice infected with *Nippostrongylus brasiliensis* expressed the pore-forming protein perforin-2 functioned as a conduit on the plasma membrane to facilitate IL-33 export contributing to mucosal immunoregulation [37]. In contrast with myeloid cells, Chen et al. [38] recently found that a neo-form murine amino-terminal p40 fragment gasdermin D promoted cytosolic IL-33 secretion by forming pores in the cell membrane without the apparent occurrence of cell death in allergen-exposed lung epithelial cells. After full-length IL-33 is released by cells under injury or stress, the serine proteases cathepsin G and elastase, released by neutrophils and mast cells, can shear full-length IL-33 to produce highly active IL-33 [39,40,41]. In addition, some proteases produced by fungi, house dust mites, bacteria, and pollens can cleave full-length IL-33 to form mature IL-33 and activate group 2 innate lymphoid cells (ILC2s) [41]. Apart from proteases, caspases also participate in the process of IL-33. The earlier studies found caspase 3 and 7-dependent proteolysis of IL-33 at D178 locus dramatically attenuated its bioactivity within apoptotic cells, most possibly by destroying the receptor-binding ability of IL-33 [27]. However, a recent study reported that IL-33 cleaved by caspases 3 and 7, which were activated by RIPK1-caspase 8 ripoptosome, at residues D175 and D178 maintained a high activity to ST2 receptor and elicited allergic inflammation [42]. The differences in IL-33 activity by caspase 3 and 7 might be due to different pathological conditions. A rare variant of human IL-33 (NM_001199640:exon7:c.487-1G>C (rs146597587-C) is associated with lower eosinophil counts and a reduced risk of asthma in Europeans; this is because rs146597587-C results in a premature stop codon that leads to the expression of truncated IL-33 (deletion of C-terminal residues 205–270), which has normal intracellular localization but cannot bind IL-33R/ST2 to activate ST2-mediated intracellular signaling [43,44]. As a result, it is necessary to determine if IL-33 cleavage variants are functional.

## 3. Nuclear IL-33 Regulates Gene Expression

Apart from its role as a cytokine, IL-33 regulates gene expression in cells. In epithelial and endothelial cells, intracellular IL-33 (icIL-33) is constitutively expressed and binds to chromosomes to regulate gene expression via multiple pathways: (1) icIL-33 binds to nucleosome acidic patches on the histone H2A-H2B dimer in heterochromatin to regulate chromatin structure and gene expression; (2) nuclear IL-33 may serve as a transcription factor that binds to the transcriptional repressor histone methyltransferase SUV39H1 to downregulate expression of soluble IL-1R4 and IL-6; and (3) icIL-33 binds to the transcription factor nuclear factor-κB (NF-κB) to block NF-κB activity and limit proinflammatory signaling [3,6,9,45,46,47]. The knockdown of icIL-33 in KKU-055 cholangiocarcinoma cells using shIL-33 resulted in increased cell proliferation and invasion, with upregulation of both NF-κB and IL-6 in 3D culture compared to control cells [48]. However, treatment with rhIL-33 also promoted KKU-055 cell proliferation by inducing NF-κB and IL-6 expression [48]. These results indicated that intracellular and extracellular IL-33 play distinct regulatory roles in cholangiocarcinoma. A recent study showed that IL-33 induced by an oncogenic H-Ras mutant (H-Ras (G12V)) was mainly located in the nuclei of NIH-3T3 cells, and Ras (G12V)-induced cyclin D1 protein synthesis was significantly suppressed by IL-33 knockdown [49], suggesting a novel role for icIL-33 in cellular transformation. Genetic deletion of IL-33 in retinal endothelial cells reduced pathological retinal neovascularization [50]. IL-33 enhanced de-ubiquitination and stabilizes the notch1 intracellular domain via interactions with BRCA1-associated protein 1 and Numb in human retinal microvascular endothelial cells, as well as in a murine model of oxygen-induced retinopathy [50]. A recent study reported that icIL-33-mediated activation of the Smad signaling pathway in epithelial cells was essential for cancer development during chronic inflammation [51]. Thus, icIL-33 resides in the nucleus and regulates the expression of a wide range of genes. However, the detailed mechanisms by which IL-33 regulates gene expression remain to be explored. It should be noted and emphasized that in a series of elegantly designed experiments [52,53] recently discussed by Cayrol et al. [13], the authors found no evidence to support a role for icIL-33 in regulating gene expression in epithelial cells. Thus, the cell-intrinsic nuclear roles of IL-33 in various cell types remain to be clarified in future work.

## 4. ST2 Expression in Immune Cells

Extracellular IL-33 binds to its transmembrane receptor, ST2, and its co-receptor, IL-1RacP, to mediate biological functions in various diseases [8,9,10]. ST2 is mainly expressed in a variety of innate and adaptive immune cells, including Th1 cells, Th2 cells, regulatory T (Treg) cells, CD8^+^ T cells, B cells, ILC2s, natural killer (NK) cells, monocytes, macrophages, mast cells, neutrophils, basophils, and eosinophils (Figure 1) [5,54,55]. In contrast to the continuous ST2 expression in Th2 cells, the expression of ST2 in Th1 cells is tightly controlled and transient upon differentiation in vitro and in vivo during lymphocyte choriomeningitis virus (LCMV) infection, and the expression depends on the transcription factors T-bet and STAT4 [56]. IL-12 induces ST2 expression in Th1 cells and CD8^+^ T cells [16,57]. This evidence indicates that IL-33 can directly enact a wide range of regulatory functions in immune and non-immune cells. It is now known that the IL-33/ST2 axis can generate a protective or deleterious immune response and regulates the body homeostasis based on the targeting cells and local microenvironment.

## 5. Negative Regulation of the IL-33/ST2 Axis

Soluble ST2 (sST2) is the most important antagonist of IL-33 identified so far. As a soluble receptor, sST2 binds to IL-33 to prevent ST2–IL-33 binding, consequently blocking downstream signaling and immune response. For example, in a mouse model of allergic airway inflammation, sST2 reduced production of IL-4, IL-5, IL-13, and other cytokines [58]. In addition, the single immunoglobulin IL–1R-related molecule (SIGIRR) impacts the dimer structure of ST2 and IL-1RAcp, weakening signaling downstream of ST2 [59]. Active IL-33 is rapidly oxidized (within hours) at four cysteine residues located in its C-terminal domain (Cys208, Cys227, Cys232, and Cys259) to induce the formation of two disulfide bridges and trigger a conformational change that blocks its receptor binding ability and leads to inactivation [60]. IL-13 inhibits ST2 and sST2 expression, as shown in IL-13 knockout mice, confirming the negative regulatory role of IL-13 on the IL-33/ST2 signal pathway [61]. Besides, ST2 internalization regulated by glycogen synthase kinase 3β or focal adhesion kinase attenuates IL-33-induced cytokine release [62]. The apoptotic caspases 3 and 7 cleave human IL-33 at amino acid D178 and/or the D175GVD178 consensus site, generating two inactive fragments unable to bind ST2 [27,28], although a recent study argues against this conclusion in an infection model [42]. These approaches promise to further our understanding of the range and duration of IL-33/ST2 activity.

## 6. Effects of IL-33/ST2 on Immune Cells

IL-33 mediates various biological functions by acting directly on various innate and adaptive immune cells. 

### 6.1. Effects on DCs 

IL-33 increases the expression of mature DC markers such as CD80 and CD40, pro-inflammatory cytokines such as IL-4, IL-5, IL-13, tumor necrosis factor (TNF)-α and IL-1β, and chemokines such as C-C motif chemokine ligand 17 (CCL17) [63]. IL-33-activated DCs promote naïve T cell differentiation into Th2 cells [64], whereas IL-33-stimulated immature DCs generated IL-2 and amplified Treg cells [65]. Additionally, DCs can produce IL-33 and are simultaneously stimulated by IL-33 to generate a positive feedback regulatory loop [63].

### 6.2. Effects on Macrophages 

IL-33 increases the expression of TLR-4, soluble CD14, myeloid differentiation protein 2 (MD2), and myeloid differentiation primary-response protein-88 (MyD88) in mouse macrophages in vitro to enhance the inflammatory response to LPS [66]. On the other hand, IL-33/ST2 signaling is involved in alternative activation of type 2 macrophage (M2) polarization. IL-33 induces the recruitment of M2-like macrophages to the tumor and stimulates macrophages to express M2 markers and produce matrix metalloproteinase-9, prostaglandin E2 and other molecules, both in vitro and in vivo [67,68]. IL-33 induces M2-like macrophage polarization by activating ornithine decarboxylase, a key enzyme that catalyzes the synthesis of polyamines [69]. In contrast, another study showed that the IL-33/ST2 axis enhances cell oxidative phosphorylation during IL-4-induced M2 polarization [70]. Kurowska-Stolarska et al. reported that ST2-deficient mice exhibited attenuated OVA-induced airway inflammation and decreased M2 differentiation, while IL-33 altered alveolar macrophages toward an M2 phenotype, promoting expression of mannose receptor and IL-4Rα and production of CCL24 and CCL17 in an IL-13-dependent manner [71]; these results indicate that the IL-33-ST2 axis may be involved in M2 differentiation and activation during airway inflammation. Apart from a supporting role in IL-4-induced polarization, Faas et al. [72] showed that, in the resolution of inflammation upon tissue injury, IL-33 promotes a mitochondrial rewiring of macrophages and the consecutive activation of the transcription factor GATA3, which orchestrates the IL-4-independent differentiation of M2. IL-33 deletion significantly decreases mortality, as well as serum levels of IL-1β and IL-18 in cecal ligation and puncture mice, by reducing macrophage apoptosis/pyroptosis, caspase-1 expression, and NF-κB/p38MAPK signal pathway [73]. In addition, the impaired macrophage migration has been reported in IL-33-deficient mice [74].

### 6.3. Effects on Mast Cells

IL-33-stimulated mast cells produce IL-4, IL-5, IL-6, CCL4, and C-X-C motif chemokine ligand 8 (CXCL8), exhibit enhanced degranulation ability, recruit eosinophils and basophils to inflammatory sites, and activate M2 and Treg cells during allergic airway inflammation [75,76,77]. In human mast-cell lines, IL-33 enhanced production of substance P-mediated vascular endothelial growth factor [78]. On the other hand, activated mast cells produce proteases to digest peripheral connective tissue and splice full-length IL-33, enhancing IL-33 activity and leukocyte infiltration [36].

### 6.4. Effects on Granulocytes and MDSCs 

In neutrophils, IL-33 induced production of IL-4, IL-5, IL-9, and IL-13 in a time- and dose-dependent fashion, promoting allergic airway inflammation in mice [79,80]. IL-33 also promotes the formation of neutrophil extracellular traps (NETs) in models of infection and IR [81,82]. In addition, the IL-33/ST2 axis maintains eosinophil survival through autocrine granulocyte macrophage colony-stimulating factor (GM-CSF) [83]. In eosinophils, IL-33-activated eosinophils can gain cytotoxic functions against tumor cells by directly killing tumor cells and by indirectly affecting immune regulatory pathways and are involved in restricting tumor growth in mouse models of colorectal cancer [83,84,85]. Besides, IL-33 induced a direct tumor-killing effect by enhancing the expression of effector molecules, including degranulation markers (CD63 and CD107a), activators (CD69), adhesion molecules (CD11b/CD18 and ICAM-1), cytokines (TNF-α), and effector molecules (granzyme A) [85,86,87]. In basophils, IL-33 increases CD11b expression, promotes degranulation in response to IgE-crosslinking, and enhances adhesiveness and eotaxin-induced migration in human basophils [88]. IL-33-treated human basophils produce pro-inflammatory cytokines, including IL-1β, IL-4, IL-5, IL-6, IL-8, IL-13, and GM-CSF [88,89]. IL-33 induces basophils to increase expression of a degranulation marker (CD63) and granzyme B, enhancing tumor-killing ability in vitro [90]. IL-33, in synergy with IL-3, stimulates IL-9 production in human basophils [91]. IL-33 can expand myeloid-derived suppressor cells (MDSCs) during cancer progression. Exogenous IL-33 enhances systemic and intra-tumoral accumulation of CD11b^+^Gr-1^+^MDSCs with enhanced immunosuppressive activity in 4T1 breast tumor-bearing mice [92,93]. In contrast, IL-33 decreases the accumulation of MDSCs with reduced immunosuppressive ability in the spleen and tumor in a B16 melanoma-bearing mouse model [94,95]. These contradictory observations may arise from differences in tumor type or microenvironment, which should be further clarified in the future.

### 6.5. Effects on NKs and ILC2s 

IL-33 enhances NK cell aggregation at inflammatory sites and promotes the production of inflammatory cytokines TNF-α and IFN-γ [76,96]. IL-33 and IL-12 synergistically induce secretion of IFN-γ, TNF, and GM-CSF in human NK cells through the p38 MAPK pathway [89,97]. IL-33 enhances production of both IL-4 and IFN-γ by human Vα24^+^ iNKT cells in a dose-dependent manner in the presence of alpha-galactosylceramide antigens [89]. IL-33 preferentially acts on ILC2s by promoting their expansion, recruitment, and activation [98]. IL-33 stimulates ILC2s to produce epidermal growth factor-like molecule amphiregulin (ARGE) and type 2 cytokines, including IL-4, IL-5, and IL-13, in a GATA3-dependent fashion [99,100,101,102,103]. Interestingly, it was recently shown that IL-33 is required for the proliferation and activation of ILC2s and the formation of “trained” ILC2s in newborns’ lungs [104,105].

### 6.6. Effects on T Cells 

Generally, IL-33 induces Th0 cells to differentiate into Th2 cells as controlled by STAT5 [106,107]. IL-33 enhances production of IL-5, IL-13, and IFN-γ in a Th2 cell-polarizing culture system during antigen-dependent and -independent T cell responses in humans [89]. ST2-deficient Th1 cells produce less TNF-α and INF-γ. IL-33 promotes the antiviral capability of Th1 cells [56]. These results suggest that IL-33 may amplify both Th1 and Th2 immune responses. IL-33, in combination with transforming growth factor-β (TGF-β), enhances the capacity of CD4^+^ T cells to produce IL-9 [108]. IL-33 stimulates Treg responses by enhancing TGF-β-mediated differentiation of Treg cells and providing a signal for Treg cell accumulation and maintenance in inflamed tissues [109]. IL-33 induces AREG expression in ST2^+^Foxp3^+^GATA3^+^ Treg cells to enhance Treg cell function and promote the repair of damaged tissues [110,111]. IL-33-stimulated mouse CD11c^+^ DCs are able to secrete IL-2 to selectively expand ST2^+^CD4^+^Foxp3^+^ Treg cells [65]. In mice, IL-33-deficient Treg cells exhibit attenuated immunosuppressive properties due to epigenetic re-programming that increases chromatin accessibility of the *Ifng* locus, enhancing IFN-γ production in an NF-κB-T-bet-dependent manner [112]. IL-33 induces CCL2 production in esophageal squamous cell carcinoma to recruit Treg cells [113]. Treg-specific ST2 deletion enhances tumor-infiltrating CD8^+^ T cells in a mouse lung adenocarcinoma model [114], supporting the idea that IL-33/ST2 signaling-activated Treg cells exhibit pro-tumorigenic functions. In contrast, some studies have found that IL-33 can disrupt Treg cell-mediated immunosuppressive functions by upregulating GATA3 expression and generating type 2 cytokines [64,65,77,115]. In a mouse model of choriomeningitis virus infection, IL-33 released by non-hematopoietic cells can enhance the antiviral capacity of CD8^+^ T cells [57,116]. In cooperation with IL-12 produced in response to LCMV/MCMV viral infection, IL-33 increases the expansion of activated CD8^+^ T cells and promotes secretion of antiviral cytokines, such as IL-10 and IFN-γ [117]. Many studies have reported that IL-33 upregulates programmed cell death protein 1 (PD-1) and/or PD-L1 in many tumor and immune cells, including as effector CD4^+^ T cells, CD8^+^ T cells, NK cells, and ILC2s [118,119,120]. IL-33 treatment enhances the frequency at which CD8^+^ T cells express cytotoxic T-lymphocyte-associated protein 4 (CTLA-4), PD-1, and KLRG-1 in a pulmonary metastasis mouse model [120]. In conclusion, IL-33 plays important and complex roles in the T cell-mediated immune response and Treg cell function in various diseases.

### 6.7. Effects on B Cells 

Studies on IL-33 in B cells have been limited to date. Using mixed bone marrow chimeric mice, Stier et al. found that IL-33 deficiency enhances the frequency of B cell growth, starting at the pro-B cell stage, via a cell-intrinsic, ST2-independent mechanism [121]. In vitro and in vivo IL-33 significantly enhances the activation and cell proliferation of mouse B1 cells, as well as the production of IgM, IL-5, and IL-13, in an ST2-dependent manner; therefore IL-33 treatment significantly exacerbates oxazolone-induced contact sensitivity in mice [122]. On the other hand, IL-33 stimulates B cells to generate IL-10-producing CD19^+^CD25^+^CD1d^high^IgM^high^CD5^−^CD23^−^Tim-1^-^ regulatory B cells to protect mice from inflammatory bowel disease [123]. 

## 7. Extracellular IL-33-Induced Signaling Pathways

**Figure 2:**
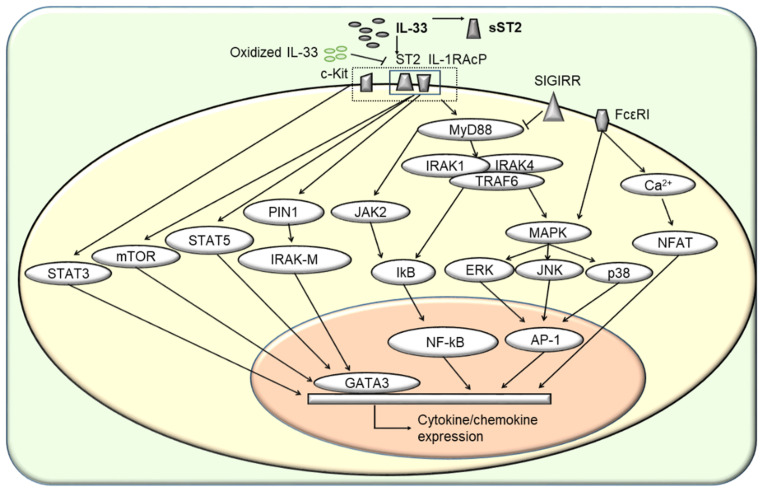
The intracellular signaling pathway of IL-33R. When IL-33 binds to ST2, IL-1RAcp is recruited to form a ligand-receptor complex, generating the minimum IL-33 receptor complex. The complex binds to the intracellular TIR domain and activates IRAK1, IRAK4, MyD88, and TRAF6. IRAKs induce the phosphorylation and degradation of IκB-α to generate NF-κB, subsequently activating the MAPK signaling pathway, which involves ERK1/2, P38, and JNK, and activator protein 1 (AP-1). Transcription factors NF-κB and AP-1 induce target gene expression in response to IL-33. Except for the classic shared mechanism of the IL-1R family, IL-33 interacts with IL-2, IL-7, and TSLP to mediate gene expression in a STAT5-dependent manner. In mast cells, the formation of this receptor complex also requires the presence of c-kit. FcƐR1 and ST2 jointly activate nuclear factor of activated T cells (NFAT) by mobilizing intracellular Ca^2+^. In mouse peritoneal macrophages, the activation of NF-κB induced by IL-33 is dependent on JAK2. IL-33 mediates various cytokine or chemokine expressions through classic and exclusive signaling pathways in different immune cells, such as IL-4, IL-5, and IL-13 in Th2 cells, CCL4, CXCL8, IL-6, IL-8, and IL-13 in mast cells, which have been summarized in Figure 1. sST2 is the most critical antagonist of IL-33 identified so far.

Recently, it has become clear that the minimal IL-33 receptor complex consists of ST2 and IL-1RAcp. ST2 can be divided into transmembrane ST2 (ST2L) and sST2 [124]. ST2 will be used to refer to ST2L throughout the rest of this manuscript. sST2 blocks IL-33/ST2 signaling because it lacks a transmembrane region and intracellular structural domain. When IL-33 binds to ST2, the conformation of ST2 changes, and IL-1RAcp is recruited to form a ligand-receptor complex. This mediates activation of a downstream signaling pathway in which ST2 is the specific receptor and IL-1RAcp is the common receptor [125,126,127]. After IL-33 binds to the heterogeneous dimer receptor on the membrane, the intracellular cytoplasmic Toll/IL-1R (TIR) domain is activated, subsequently activating MyD88, IL-1 receptor-associated kinase (IRAK), and TNF receptor-associated factor-6 (TRAF6) signaling pathways [128,129]. TRAF6 then induces the phosphorylation and degradation of IκB-α to generate NF-κB, subsequently activating the MAPK signaling pathway, which includes extracellular signal-regulated kinase (ERK1/2), P38, and c-Jun N-terminal kinase (JNK), and AP-1. The transcription factors NF-κB and AP-1 induce target gene expression (Figure 2). IL-33, combined with IL-2, IL-7, and TSLP, mediates type 2 cytokine generation in a STAT5-dependent manner in ILC2s [130]. In mast cells, the formation of the receptor complex also requires the presence of receptor tyrosine kinase c-kit [131], which can activate STAT3, ERK1/2, protein kinase B, and JNK1 and promote IL-6 production. In addition, FcƐR1 and ST2 jointly activate the nuclear factor of activated T cells (NFAT) through the mobilization of Ca^2+^ [1,54,131]. During helminth infection, ST2 forms an active signaling interaction with epidermal growth factor receptor on Th2 cells to activate the MAPK signaling pathway, phosphorylate ERK, and increase IL-13 production [132]. In mouse peritoneal macrophages, NF-κB activation induced by IL-33 is dependent on Janus kinase 2 (JAK2) [133]. IL-33 can activate members of the mTOR pathway, such as phosphoinositide-3 kinase, in Th2 cells, macrophages, and eosinophils [134]. In human monocytes, IL-33 treatment induces production of reactive oxygen species, decreases production of the M1-related cytokines CXCL10 and TNF-α, and increases production of the M2-related cytokine CCL22, likely through the AMP-activated protein kinase activation and mitophagy pathway [135]. IL-33 activates DCs through a signaling axis involving the prolyl cis-trans isomerase PIN1-IRAK-M, after which the DCs polarize T cells to Th2 cells [136]. It was recently reported that salt-inducible kinases (SIKs), including SIK2 and SIK3, are required for IL-33-stimulated expression of IL-13, GM-CSF, and TNF in mouse mast cells [137]. Human SIGIRR forms a complex with ST2 after IL-33 stimulation and subsequently interferes with IL1RAcP recruitment to inhibit the IL-33/ST2-mediated signaling pathway [59], providing the negative regulatory loop of the IL-33/ST2 pathway.

Currently, more and more Omics-based studies are applied to explore potential signaling network. Pinto et al. [138], carrying out a quantitative phosphoproteomic analysis, reported that the phosphorylation of multiple protein kinases and several protein phosphatases that are induced by IL-33, including mitogen-activated protein kinase-activated protein kinase 2 (Mapkapk2), receptor-interacting serine-threonine kinase 1 (Ripk1), NAD kinase (Nadk), and protein tyrosine phosphatase, non-receptor type 12 (Ptpn12), and inositol polyphosphate-5-phosphatase D (Inpp5d), which have not been reported previously. The results of functional analysis suggested IL-33-induced Rho-dependent signaling. Further, this research group applied quantitative temporal phosphoproteomics analysis and identified several kinases and phosphatases regulated across timepoints; they found that IL-33 regulated phosphorylation sites on transcription factors, which revealed several cellular processes of IL-33 activation, including leukocyte adhesion, response to reactive oxygen species, cell cycle checkpoints, and DNA damage and repair pathways [139]. As technology advances, a more detailed and complete IL-33 signaling network will be achieved. 

## 8. IL-33 and Immune-Related Diseases

IL-33 knockout mice can thrive under sterile conditions without noticeable phenotypic abnormalities. However, when inflammation occurs, the immune responses of IL-33 knockout mice are abnormal compared to those of wild-type mice [21,140]. IL-33 is involved in various immune and inflammatory diseases and is highly relevant to infections, transplantation, and cancer. It should be noted that, although IL-33 is closely involved in inflammatory processes, it also contributes to many processes beyond immune functions, including metabolism, tissue homeostasis and repair, and development [10,141,142,143].

### 8.1. Inflammatory and Autoimmune Diseases 

Numerous studies have demonstrated the importance of IL-33 in airway inflammation [7,8,10,144,145]. In clinical practice, serum IL-33 levels are positively correlated with the severity of allergic asthma, and IL-33 concentrations have been used to assess the severity of allergic asthma [23,146]. In airway inflammation, IL-33 plays vital roles in various cells, including eosinophils, macrophages, DCs, and Th2 cells [106]. IL-33 activates ILC2s and Th2, producing type 2-related cytokines, activating eosinophils and polarizing M2 [5,71,136,147,148,149,150,151,152,153]. It has been recently reported that infiltrated neutrophils promote *Alternaria alternata*-induced airway inflammation through IL-33 cleavage in mice [154]. In a mouse model of airway inflammation, both glucagon-like peptide 1 receptor and *H. polygyrus* Alarmin Release Inhibitor (HpARI) can suppress the release of IL-33, the recruitment of eosinophils and ILC2s, and the production of type 2 cytokines [155,156]. Anti-IL-33R mAb and sST2 can effectively treat allergic asthma and are expected to be promising drugs for the treatment of allergic asthma [153,155,156,157]. In airway inflammation, Phase 1 and 2 clinical trials using an anti-IL-33 mAb, itepekimab, in patients with moderate to severe asthma showed decreased blood eosinophil levels and improved lung function, suggesting that IL-33 may be a promising molecular target for the treatment of allergic asthma [158,159,160]. In the patients with seasonal allergic rhinitis, the serum level of IL-33 was found to be significantly increased and there is a significant association between susceptibility to allergic rhinitis and IL-33 polymorphism. The elevated serum level of IL-33 not only induces the inflammatory response, but also its concentration is positively correlated with allergic rhinitis severity [161]. IL-33 stimulates mast cells to increase histamine secretion under the condition of cross-linkage of FcεRI with IgE–ragweed pollen; additionally, ragweed pollen-driven endogenic IL-33 plays an essential role in the recruitment of eosinophils and basophils by inducing the production of various chemo-attractants, including eotaxin, MIP-1α, RANTES, and MCP-1 [162]. Apart from that, IL-33 was found to be associated with the IL-17 as well as IL-31 production in allergy-driven pathologies of allergic rhinitis [163]. These also suggest that the involvement of IL-33/ST2 axis in the type 2 response is different from the typical Th2 cytokines such as IL-4, IL-5, and IL-13 in terms of its independent role in allergic rhinitis. Except for respiratory allergies, IL-33 is predominant in ILC2-inducing type 2 cytokines in atopic dermatitis [164]. IL-33 triggers the mast cell and basophil activation and provokes the overproduction of pro-inflammatory cytokines, and it also induces the migration, maturation, adhesion, and survival of these immune cells [165]. An IL-33 injection in mice intradermally elicits a scleroderma-like reaction with an increase in dermal collagen fibers or a psoriasis-like dermatitis with a thickened epidermis [166].

In human chronic obstructive pulmonary disease, increased IL-33 concentrations in lung epithelial cells are related to disease severity. Smoking increased the expression of IL-33 in lung epithelial cells, enhances the response to virus infection, inhibits ST2 expression in ILC2s, increases ST2 expression in NK cells and macrophages, and aggravates the disease, with increased expression of TNF-α, IL-12, and IFN-γ observed in mice that were sensitized via smoking and re-infected with the virus [167].

Genotyping studies have shown that the IL-33 rs10975519 CC genotype in females is associated with a decreased risk of developing rheumatoid arthritis [168], while the IL-33 rs16924159 AA genotype correlates with higher disease activity and poorer clinical outcomes in patients with rheumatoid arthritis and ankylosing spondylitis following treatment with TNF inhibitors; this suggests that IL-33 gene polymorphisms may be potential candidate biomarkers of disease susceptibility and anti-TNF treatment response [168]. IL-33 and ST2 levels in both the serum and synovium of patients with rheumatoid arthritis, systemic sclerosis, and systemic lupus erythematosus are significantly increased, and levels are correlated with disease severity [3,169,170,171]. IL-33 treatment significantly worsens rheumatoid arthritis, whereas antagonizing IL-33 signaling decreased disease severity in a mouse collagen-induced arthritis model [172,173,174]. Antigen-induced arthritis is exacerbated by IL-33, which activates mast cells and causes them to produce proinflammatory cytokines, including IFN-γ, TNF, and IL-17 [173,174].

IL-33 expression is increased in the intestinal mucosa and barrier of patients with inflammatory bowel disease [175]. IL-33 expression levels are increased and positively correlated with disease severity in patients with ulcerative colitis and Crohn’s disease, as well as in a mouse model [175,176]. In a dextran sodium sulfate-induced acute colitis mouse model, both IL-33 and ST2-knockout mice showed delayed intestinal inflammation symptoms [140,177]. The IL-33/ST2 axis regulates the contributions of nucleotide-binding oligomerization domain-containing 2 and ILC2s to early events in Crohn’s disease pathogenesis [178]. However, contradictory results revealed a protective effect of the IL-33/ST2 axis in these inflammatory diseases, highlighting the complex roles of IL-33. A clinical study showed that mRNA expression levels of IL-33 were decreased in biopsy specimens of ulcerative colitis patients compared with the control group, and a negative correlation was observed between IL-33 expression and the severity of ulcerative colitis [179]. IL-33 treatment alleviates colitis in mouse models of Crohn’s disease by altering Th1 cells toward Th2 and Treg cells. IL-33 treatment decreases inflammatory bowel symptoms in trinitrobenzene sulfonic acid-induced colitis [180,181].

Clinical results regarding the serum levels and roles of IL-33 in systemic lupus erythematosus (SLE) patients are conflicting [182], possibly due to issues of detection efficacy related to the heterogeneity of SLE patient cohorts in terms of disease activity and stage, treatment, and genetic background. Anti-IL-33 treatment significantly reduced mouse mortality, serum anti-dsDNA levels, renal damage, and circulating immune complexes in an MRL/Lpr mouse model, likely by promoting the expansion of Treg cells and MDSCs while decreasing expression of the pro-inflammatory cytokines IL-1β, IL-6, and IL-17 [183], which suggested a protective effect of IL-33 antagonization on SLE in mice. Moreover, in a mouse model of allergic dermatitis, increased IL-33 in sensitized skin caused neutrophil infiltration and a high expression of IL-4, IL-17a, CXCL1, and CXCL2, but not IFN-γ; additionally, the proinflammatory factors promote virus replication and inflammation after incubated with vaccinia virus, which is involved in Eczema Vaccinatum [184]. The IL-33/ST2 axis promotes the development of primary Sjogren’s syndrome by activating salivary epithelial cells and the type 1 immune response in a mouse model of experimental Sjogren’s syndrome [185]. Thus, the IL-33/ST2 axis plays an essential role in inflammatory and autoimmune diseases, both in animal models and in clinic practice. 

### 8.2. Infectious Diseases 

IL-33 is closely involved in pathological process in parasite and pathogen infection. Patients infected with *Schistosoma haematobium* have higher amounts of IL-33 in their plasma [186]. Serum IL-33 levels are increased in children infected with *Plasmodium falciparum* [187]. The injection of recombinant IL-33 attenuates cerebral malaria infection in mice [188]. IL-33 increases the expression and secretion of IL-5, IL-13, and other type 2 cytokines by mast cells and ILC2s during parasitic infection in a model of intestinal parasite infection [99,189]. It also promotes parasite clearance by increasing Th2-related cytokines and reducing the production of Th1 and Th17-related cytokines [189,190]. In a lung disease model of *Pneumocystis murina*-infected mice, IL-33 enhanced alveolar macrophages to M2a polarization resulting in a more efficient destruction of *Pneumocystis murina* [191]. ST2 deficiency reduces the degree of infection in a model of *Cryptococcus neoformans*-infected lung disease [192]. *Porphyromonas gingivalis* is a critical periodontal pathogenic bacterium that can enhance IL-33 expression in human gingival epithelial cells by activating PAR-2-PLC-p38/NF-κB-signaling pathways [193]. In patients with periodontitis, IL-33 is highly expressed both at the site of onset and in a periodontitis model infected with *Porphyromonas gingivalis*, and IL-33 exacerbates alveolar bone loss and aggravates the disease in a RANKL-dependent manner [194]. In mice with *Pseudomonas aeruginosa*-induced keratitis, IL-33 effectively reduces inflammation by polarizing the macrophage production of anti-inflammatory mediators [195]. In addition, the IL-33/ST2 axis provides a protective effect against *Streptococcus pyogenes* infection by enhancing neutrophil migration and bactericidal activity [196]. In patients infected with HIV or dengue virus, the sST2 levels in sera rise [197,198]. In a respiratory syncytial virus-infected mouse model of tracheitis, the infiltration of inflammatory cells in lung tissue is reduced following treatment with ST2 neutralizing antibodies [199]. IL-33 aggravates airway inflammation induced by the H3N2 influenza A virus [200]. In a mouse model infected with LCMV, IL-33 released by non-hematopoietic cells enhances the expression of ST2 and improves the antiviral ability of CD8^+^ T cells [116]. The IL-33/ST2 axis expands and activates NK cells and DCs to promote host defenses against viral infection in mice [96,201]. Therefore, IL-33 plays an important role in various infectious diseases.

### 8.3. Cardiovascular Diseases 

Serum levels of IL-33 and sST2 are increased and correlate positively with the degree of heart failure, making them potentially useful for predicting disease severity or mortality outcomes in patients with cardiovascular diseases, probably as independent risk factors of heart failure [202,203,204]. In mice, IL-33/ST2 signal activation can effectively control myocardial hypertrophy and cardiac fibrosis. Myocardial hypertrophy and fibrosis were observed in ST2-knockout mice, and recombinant IL-33 exerted protective effects on cardiac cells [125]. Compared with wild-type mice on a high-fat diet, ST2-knockout mice had increased body weight fat content and were susceptible to pancreatic islet injury [205,206]. IL-33 prevents the formation of atherosclerotic plaques, reducing atherosclerosis [174,207,208]. In mouse models of myocardial infarction, IL-33 was found to reduce cardiac cell apoptosis and enhance cardiac survival and function [209]. Therefore, IL-33 is vital in maintaining cardiovascular system homeostasis and may be an essential indicator for predicting potential atherosclerosis.

### 8.4. Neurological Diseases 

IL-33 is highly expressed in brain tissue [21] and is indispensable to neural circuit development. In the developing brain, IL-33 produced by synapse-associated astrocytes is required for signals to microglia to promote increased synaptic engulfment and thereby fine-tune brain connectivity [11]. Apart from homeostatic development, IL-33 plays an important role in various neurological diseases, including neurodegenerative diseases, central nervous system infectious diseases, central nerve injury, and chronic pain [210,211]. Il-33, released by damaged oligodendrocytes, promotes recovery following central nervous system injury via acting on local astrocytes and microglia to induce chemokines critical for monocyte recruitment and polarization to M2 fate [212]. In an experimental mouse model of autoimmune encephalomyelitis (EAE), IL-33 played a protective role by switching a predominantly pathogenic Th17/Th1 response to Th2 activity, promoting microglia polarization toward anti-inflammatory M2 and suppressing the activation of astrocytes and microglia [213,214]. In a protozoan-induced mouse encephalitis model, iNOS, TNF, and IFN-γ levels were elevated and encephalitis increased in ST2-knockout mice [215]. In a mouse model of Alzheimer’s disease, IL-33 could reduce levels of β-amyloid (Aβ) in the brain, repair synaptic damage, and ameliorate the disease by promoting the recruitment and phagocytosis of microglia cells, as well as the secretion of anti-inflammatory factors by microglia cells and macrophages [216]. Recently, it was demonstrated that IL-33 improved Aβ pathology by reprogramming chromatin accessibility and PU.1 transcription factor binding in microglial cells in Alzheimer’s disease [217]. IL-33/ST2 reduced brain lesion size and functional deficits after traumatic brain injury, in part by enhancing Treg cell infiltration and immunosuppressive function [218]. Therefore, the protective role of IL-33 may be applied in treating neurological diseases. 

### 8.5. Tumors 

Numerous studies have demonstrated that IL-33 plays dual roles regulating tumor transformation, growth, and metastasis in many cancers, including non-small cell lung cancer (NSCLC), colorectal cancer, gastric cancer, pancreatic tumor, and breast cancer by directly acting on tumor cells or by indirectly affecting immune cells or the tumor microenvironment [219,220,221,222,223,224]. In some populations of China, individuals with at least one C allele of ST2 rs3821204 exhibited a higher risk of hepatocellular carcinoma compared to those with GG genes [225]. Patients with NSCLC in their peripheral blood exhibited increased IL-33 levels in serum and plasma, while IL-33 levels in plasma were decreased with disease progression [226,227]. Co-culturing of human NSCLC cells with IL-33 antagonists reduces M2 polarization and Treg cell accumulation, inhibiting the growth of cancer cells [220]. IL-33 can enhance glucose uptake and glycolysis and promote cell proliferation by upregulating the expression of glucose transporter 1 in NSCLC cells [228]. In colorectal cancer (CRC), IL-33 levels were increased in cancer tissues, and IL-33 was shown to promote tumor growth and liver metastasis [229,230]. Under the action of IL-33, the growth and metastasis rate of CRC in mice was accelerated [231]. After injection of IL-33 into human CRC-bearing nude mice, the tumors grew rapidly, and the expression of IL-6, CXCR4, matrix metallopeptidase 2 (MMP2), and MMP9 was increased [232]. The blockade of IL-33 and/or ST2 results in tumor growth inhibition accompanied by the reduced accumulation of tumor-promoting Treg cells, M2-like macrophages, and IL17RB^+^ILC2s [220,233,234,235]. As an antagonist of IL-33/ST2, sST2 plays a negative role in CRC tumor growth by inhibiting IL-33-mediated angiogenesis, Th1 and Th2 responses, macrophage infiltration, and M2a polarization [231,236]. IL-33 was shown to be upregulated in metastases-associated fibroblasts in mouse models of spontaneous breast cancer metastasis and in breast cancer patients with lung metastasis. Upregulation of IL-33 instigates type-2 inflammation in the metastatic microenvironment, and mediates recruitment of eosinophils, neutrophils, and inflammatory monocytes to lung metastases, demonstrating a modulating role of IL-33 in immune microenvironment [237]. However, in B16 tumor-bearing mouse models, IL-33 promotes the infiltration of NK cells and CD8^+^ T cells and increases IFN-γ and perforin expression to kill tumor cells and inhibit tumor growth [238]. Nuclear and secreted IL-33 regulates chemokine expressions to recruit and activate circulating and resident innate immune cells, creating a pro-tumorigenic environment. Conversely, loss of nuclear IL-33 dramatically suppresses glioma growth and increases survival [239]. Park et al. found that nuclear IL-33-mediated activation of SMAD signaling pathway in epithelial cells is essential for skin cancer development in chronic inflammation [51]. A new study employing a squamous cell carcinoma murine model reported that tumor-initiating cells promote the release of IL-33 to facilitate differentiation of TGF-β-producing macrophages, upregulating IL-33 expression and increasing the invasive and drug-resistant properties of tumor-initiating cells to form a regulatory feedback loop that promotes cancer progression [240]. IL-33 promotes the production of IL-6 and MMP-3 by the ERK1/2 signaling pathway and enhances tumor invasion and escape ability in human gastric cancer (GC) cell lines [241]. It was found that IL-33 promotes cell escape and prevents platinum-induced cell apoptosis through the JNK signaling pathway in GC [242]. Tristetraprolin can effectively reduce IL-33 expression and tumor proliferation, metastasis, and escape in human GC cell lines and GC-bearing mice [243]. 

Meanwhile, IL-33 can exhibit opposite effects under different conditions. Endogenous IL-33 promotes effector CD4^+^ and CD8^+^ T cell activation and IFN-γ production to enhance antitumor responses and suppress cancer growth and metastasis in mouse models of colon and hepatocellular carcinoma [244,245,246]. IL-33 expression in the tumor cytoplasm of patients with cervical cancer is positively correlated with infiltration of CD3^+^ T cells, CD8^+^ T cells, and PD-L1 expression in tumor tissues. High IL-33 expression in tumor tissues has been associated with improved prognosis [247]. It has been reported that intracellular and extracellular IL-33 play distinct mechanistic roles; intracellular IL-33 attenuates extracellular IL-33-induced cholangiocarcinoma cell proliferation and invasion via NF-κB and GSK-3β pathways [48]. IL-33 induces DCs to express semaphorin 4A, which is essential for the upregulation of IFN-γ production by tumor-infiltrating CD8^+^ T cells and the potent antitumor effects of IL-33 [248]. Endogenous IL-33/ST2 signaling enhances tumor-infiltrating ILC2s and CD8^+^ T cell-mediated cancer immunity in pancreatic ductal adenocarcinoma-bearing mouse models [118]. In mouse models, administration of IL-33 induces DC activation to enable cross-priming of tumor-reactive CD8^+^ T cells to inhibit tumor growth [249,250,251]. IL-33 inhibits tumor growth and lung metastasis in many mouse models by recruiting and activating eosinophils [85,94,252]. Apart from solid tumors, in a murine acute myeloid leukemia model, administration of IL-33 significantly inhibited leukemia growth and improved mice survival rate in a CD8+ T cell dependent manner [253]. Therefore, the IL-33/ST2 axis exerts pro- and anti-tumorigenic effects depending on expression levels, tumor and cell types, and the microenvironment. Notably, an increasing number of recent studies have reported a synergistic effect of IL-33 in improving the efficacy of anti-CTLA-4/anti-PD-1 immunotherapy, probably because IL-33 treatment upregulates CTLA-4, PD-1, and/or PD-L1 expression in effector CD4^+^ T cells, CD8^+^ T cells, NK cells, and ILC2s [118,119,120]. It may be of great significance to explore the use of IL-33 therapy in targeted tumors to inhibit tumor growth and metastasis.

### 8.6. Transplantation 

In transplantation, it is well appreciated that there are many alarmins in grafts because of IR injury. Numerous proinflammatory alarmins have also been identified, such as ATP, mitochondrial contents, and high-mobility group box 1. However, not all alarmins are proinflammatory and some may actually have a beneficial function, such as IL-33 [254]. Levels of sST2 in sera and IL-33/ST2 expression in kidney tissues were higher in transplanted patients with acute antibody-mediated rejection, acute cell-mediated rejection, and chronic antibody-mediated rejection compared to recipients without rejection or to healthy controls [255]. Higher ST2 or IL-33 expression may be associated with chronic allograft dysfunction and acute rejection [255,256,257]. Elevated serum sST2 levels are correlated with heart transplantation rejection, as well as an increased risk for antibody-mediated alloreaction [258,259,260,261,262]. Among patients receiving kidney transplantation, serum levels of IL-33 were higher in chronic allograft dysfunction patients compared to patients with stable graft function [257]. The research on cardiac IR and IR-induced myocardial injury found that IL-33 may have beneficial effects by suppressing inflammatory cytokines’ expression and myocardial apoptosis, and increasing production of Th2 type cytokines and an upset in the balance of Th1/Th2 responses during heart transplantation [263,264]. Donor mouse cardiac allografts deficient in IL-33 exhibited dramatically accelerated vascular occlusion and subsequent fibrosis, which was accompanied by local proinflammatory iNOS^+^ macrophage augmentation [265]. In this paradigm, local administration of IL-33 prevents chronic rejection of IL-33-deficient cardiac transplants [265]. IL-33 administration increases functional CD11b^+^Gr-1^int^ MDSCs, CD4^+^Foxp3^+^ Treg cells, and Th2 responses, while simultaneously decreasing CD8^+^IFN-γ^+^ cells, ultimately resulting in significant graft prolongation in a fully allogeneic heart transplantation mouse model [266,267,268]. In addition, IL-33 has beneficial effects on prolonging allograft survival during chronic cardiac rejection through multiple mechanisms, including promoting accumulation of Treg cells and MDSCs, reducing numbers of B220^+^CD19^+^B cells, increasing production of IL-5, IL-10, and IL-13, and decreasing alloantibody and IL-17A production [267]. Guo et al. recently reported that IR injury upregulated IL-33 expression, which induced IL-5 production by graft-resident ILC2s and contributed to eosinophil infiltration into the allograft, facilitating lung allograft acceptance in a murine lung transplant model [269]. These findings indicated a potential beneficial role of IR injury for allograft survival by promoting IL-33/IL-5/eosinophil-mediated immune tolerance. Although IL-33-induced Treg cells did not show any detectable alterations in immunosuppressive capacity in vitro, the Treg cells displayed enhanced regulatory activity in vivo, promoting long-term skin allograft survival. A mechanistic study showed that enhanced expression of graft-homing chemokine receptors in IL-33-expanded Treg cells might be partially responsible for their superior suppressive activity in vivo [270]. In a mouse model of acute graft-versus-host disease (GVHD), IL-33 can reduce the GVHD response by amplifying Treg cells [271]. However, Dwyer et al. [272] reported that IL-33 promoted IL-12-independent T bet expression and Th1 cell polarization in response to alloantigens by augmenting the TCR-associated signaling pathways and inhibiting the expression of regulatory molecules, such as IL-10 and Foxp3, in a GVHD mouse model. The difference might be caused by a different detection location of GVHD model. Dwyer et al. [272] emphasized the IL-33 role in the secondary lymphoid organs, but not others, such as barrier tissues or GVDH target organs. GVHD lethality and TNF-α production are reduced in IL-33-deficient recipients, and IL-33 administration during peak inflammatory response worsened GVHD in an allogeneic-hematopoietic cell transplantation model [273]. Blocking IL-33/ST2 interactions by ST2-Fc infusion markedly reduces GVHD lethality [273], indicating that IL-33/ST2 is involved in GVHD pathogenesis. Thus, IL-33 may mainly mediate anti-graft immune response to increase graft survival. The detailed roles of IL-33 signaling in allograft rejection should be studied in the future. 

### 8.7. Other Diseases 

IL-33 is essential in maintaining skeletal muscle system homeostasis. In human tendon lesions, tendon cells that are under pressure release IL-33 [274]. IL-33 inhibits bone absorption and osteoclast differentiation in mice and humans [275]. Therefore, targeted IL-33 therapy has many potential applications in skeletal muscle system diseases. In damaged skin tissue, IL-33 is released from epithelial cells to promote wound healing by activating ILC2s [276]. IL-33/ST2 is involved in various ocular diseases, including allergic eye disease, dry eye disease, uveitis, vitreoretinal disease, and neuromyelitis optica spectrum disorder [277,278], as reviewed by Qian et al. [279]. IL-33 also plays an important role in metabolic diseases, such as obesity and type 2 diabetes, which has been summarized by Tu et al. [280].

## 9. Conclusions

Since its discovery in 2005, IL-33 has been shown as playing an important roles in many diseases. Under normal conditions, IL-33 exists mainly in the nuclei of endothelial cells, epithelial cells, and other cell types. However, it is released when cells are under stress or upon external stimulation. IL-33 acts directly on various immune and non-immune cells as a vital alarmin and proinflammatory factor, playing important immune regulatory roles and diverse biological functions that include: (1) mediating type 2 immune response, as IL-33 can stimulate mast cells, neutrophils, ILC2s, naive T cells, and other immune cells to secrete the type 2 cytokines IL4, IL-5, and IL-13, among others; (2) enhancing Treg cell immunosuppressive function; (3) promoting ILC2s to produce AREG for tissue repair; (4) directly promoting tumor growth. However, some contradictory results have been obtained from different disease models, possibly due to differences in cell types, microenvironments, and relevant molecular mechanisms, which need to be clarified in the future. The diverse biological functions and relevant molecular mechanisms of IL-33 also need to be further studied. Notably, IL-33 and sST2 can be used to monitor the severity and progression of many diseases. Nevertheless, given that IL-33 is a pleiotropic cytokine, a detailed understanding of the distinct and complicated roles of IL-33 in epithelial, stromal, and immune cell subsets is important for the development of more effective therapeutic approaches for related diseases. 

## Data Availability

Not applicable.

## Abbreviations

Aβ	β-amyloid
AP-1	activator protein 1
AREG	amphiregulin
BTK	Bruton’s tyrosine kinase
CCL	C-C motif chemokine ligand
CRC	colorectal cancer
CTLA-4	cytotoxic T-lymphocyte-associated protein 4
CXCL	C-X-C motif chemokine ligand
CXCR	C-X-C motif chemokine receptor
DAMP	damage-associated molecular pattern
DCs	dendritic cells
EAE	experimental autoimmune encephalomyelitis
ERK	extracellular signal-regulated kinase
GC	gastric cancer
GM-CSF	granulocyte macrophage colony-stimulating factor
GVHD	graft-versus-host disease
icIL-33	intracellular IL-33
IFN-γ	interferon-γ
IL	interleukin
IR	ischemia/reperfusion
IL-1F	IL-1 family
IL-1R	IL-1 receptor
IL-1Racp	IL-1 receptor accessory protein
ILC2s	group 2 innate lymphoid cells
iNKT cell	invariant natural killer T cell
IRAK	IL-1 receptor-associated kinase
JAK2	Janus kinase
JNK	c-Jun N-terminal kinase
LCMV	lymphocyte choriomeningitis virus
LPS	lipopolysaccharide
MAPK	mitogen-activated protein kinase
MD2	myeloid differentiation protein 2
MDSCs	myeloid-derived suppressor cells
MMP	matrix metallopeptidase
MyD88	myeloid differentiation primary-response protein-88
NETs	neutrophil extracellular traps
NFAT	nuclear factor of activated T cells
NF-κB	nuclear factor-κB
NK cells	natural killer cells
NSCLC	non-small cell lung cancer
PD-1	programmed cell death protein 1
SIGIRR	single immunoglobulin IL–1R-related molecule
SIK	salt-inducible kinase
SLE	systemic lupus erythematosus
sST2	soluble ST2
ST2	suppression of tumorigenicity 2
STAT	signal transducer and activator of transcription
TGF-β	transforming growth factor-β
TLRs	Toll-like receptors
TNF-α	tumor necrosis factor-α
TRAF6	TNF receptor-associated factor-6
TSLP	thymic stromal lymphopoietin
